# Polymorphisms of the *ELANE* Gene Promoter Region in End-Stage Chronic Kidney Disease Patients

**DOI:** 10.3390/genes7050017

**Published:** 2016-04-29

**Authors:** Rafael Fernandes, Bruno Freitas, Vasco Miranda, Elísio Costa, Alice Santos-Silva, Elsa Bronze-da-Rocha

**Affiliations:** 1 UCIBIO/REQUIMTE, Laboratório de Bioquímica, Departamento de Ciências Biológicas, Faculdade de Farmácia, Universidade do Porto,4050-313 Porto, Portugal; rqf@ua.pt (R.F.); brunofreitas2006@hotmail.com (B.F.); emcosta@ff.up.pt (E.C.); assilva@ff.up.pt (A.S.-S.); 2 Frenesius Medical Care, Nephrocare Maia, SA, Rua Altos 70, Maia, 4470-235 Porto, Portugal; mail@vascomiranda.com

**Keywords:** elastase, end-stage renal disease, single nucleotide polymorphism, duplication

## Abstract

End-stage renal disease (ESRD) patients have a high mortality rate that exceeds that of non-ESRD population. The hemodialysis procedure induces neutrophil activation and elastase release, which might have a role in the inflammatory process and in the development of oxidative stress. The *ELANE* gene encodes the neutrophil elastase. We analyzed the effect of *ELANE* promoter region polymorphisms and its relation with the circulating levels of elastase, as well as several clinical, biochemical and inflammatory markers in 123 ESRD patients. We found two duplications in heterozygosity in the promoter region and a new polymorphism, the c.-801G>A. ESRD patients heterozygous for the c.-903T>G polymorphism had no changes in the circulating levels of elastase or other evaluated variables, and those homozygous for the c.-741G>A polymorphism showed significant effects on neutrophils count, as well as in neutrophils/lymphocytes ratio, which might be associated with an increased inflammatory process.

## 1. Introduction

End-stage renal disease (ESRD) is a growing public health problem with an increasing worldwide prevalence. Inflammation is a common feature in ESRD patients under hemodialysis (HD) [1,2]; however, the mechanisms/factors triggering the inflammatory process are still poorly clarified. The HD procedure induces neutrophil activation and elastase release, which might be important in the inflammatory process and in the development of oxidative stress, two factors that increase the anemia and the risk of cardiovascular diseases [1,2,3]. Neutrophil elastase, a protease able to degrade several extracellular matrix proteins, is encoded by *ELANE* gene. Polymorphisms of *ELANE* gene have been associated with the development of several pathologies [4,5,6]. Indeed, the presence of mutations and single nucleotide polymorphisms (SNPs) in the codifying region and in the six repetitive tandem motifs of the promoter appear to influence the level of elastase expression, promoting proteolytic disturbances [5]. 

This study aimed to identify polymorphisms of the *ELANE* promoter region and assess their impact in the plasma levels of elastase, clinical and hematological data, iron metabolism, dialysis efficiency, inflammatory and nutritional markers.

## 2. Experimental Section

### 2.1. Patients

We performed a cross-sectional study with 123 ESRD patients (69 males and 54 females, mean [±SD] age: 65.3 [±13.9] years) on regular HD. Patients were under therapeutic HD three times per week for 3–5 h, for a median time of 2.5 (1.2–5.2) years. High-flux polysulfone FX-class dialyzer of Fresenius (Bad Hamburg, Germany) was used for the HD procedure. The main causes of renal failure were diabetic nephropathy (*n* = 66), hypertensive nephrosclerosis (*n* = 21) and other causes (*n* = 36); patients with autoimmune diseases, malignancy, and acute or chronic infection were excluded. All participants gave their written informed consent to participate in this study, previously approved by the Ethics Committee from the dialysis clinic. 

### 2.2. Methods

The *ELANE* promoter was screened in all patients using PCR-direct sequencing with forward (5’-GGAAGGACCAGAGAAGTGC-3’) and reverse (5’-CTGCCAAACCTAGACCTGAG-3’) primers, which amplify a 397bp DNA fragment. Sequencing was performed by automatized Sanger method (GATC Biotech^®^ facilities) and electropherograms were analyzed with Chromas lite 2.1.1™ software (Technelysium, Australia). Plasma elastase quantification was done by ELISA (Human PMN elastase platinum ELISA, eBioscience, Austria). Each polymorphism was statistically evaluated and correlated with clinical and dialysis adequacy markers (age, gender, type of vascular access, presence of diabetes, dialysis time, urea reduction ratio, Kt/V, creatinine, darbopoeitin dose), hematological data (hemoglobin, hematocrit, erythrocyte, MCV, MCH, MCHC, RPI, leukocyte, neutrophil, lymphocyte and reticulocyte counting, neutrophil/lymphocyte ratio), inflammatory (*C*-reactive protein, interleuquin-6 (IL-6), oxidized low-density lipoprotein (oxLDL), elastase and elastase/neutrophil ratio), nutritional (serum albumin and body mass index) and iron metabolism (iron, transferrin, transferrin saturation, ferritin and soluble transferrin receptor (sTfR) serum levels) markers, according to the methods previously described [7]. Statistical analysis was performed using the Statistical Package for Social Sciences (SPSS) version 21.0 for Windows (SPSS Inc., Armonk, NY, USA). The distribution of continuous variables was analyzed using the Kolmogorov-Smirnov test. The values of variables with normal distribution are presented as mean ± standard deviation, and those without normal distribution are expressed as median (interquartile range). According to the type of distribution, parametric or non-parametric for the comparisons between groups we used Student’s *t* test or the Mann-Whitney test, respectively. A comparison of multiple variables between groups was performed using one-way ANOVA algorithm with the post hoc Tukey test. The association between categorical variables was analyzed using the test χ^2^ or Fischer’s exact test. Differences between groups were considered statistically significant at *p* < 0.05. Moreover, the adjustment of *p* value derived from multiple statistical tests was performed for the inflammatory associated variables (CRP, IL-6, OxLDL, elastase; elastase/neutrophil ratio, albumin, neutrophils, leucocytes, neutrophil/lymphocyte ratio) using the Bonferroni correction test and a *p* < 0.006 was considered as statistically significant.

## 3. Results and Discussion

**Figure 1:**
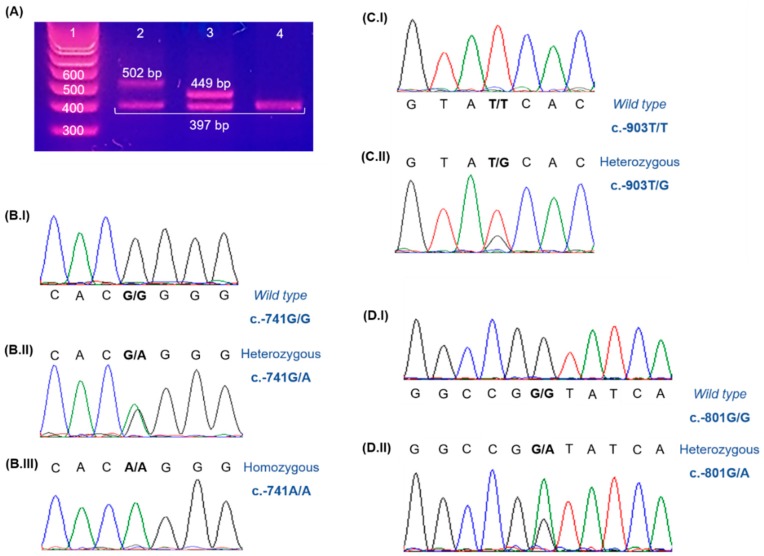
Agarose gel electrophoresis of the PCR (Polymerase Chain Reaction) products of patients presenting two bands in comparison with the PCR product of a patient with one band (**A**): lane 1, molecular weight standard; lane 2, patient with an extra block of repetitions; lane 3, patient with an extra 52bp; lane 4, patient without duplications. Electropherograms representing the identified SNPs in the studied ESRD population: wild type gene (**B.I**); heterozygous (**B.II**) and homozygous (**B.III**) for c.-741G>A polymorphism; wild type gene (**C.I**) and heterozygous (**C.II**) for c.-903T>G polymorphism; wild type gene (**D.I**) and heterozygous (**D.II**) for c.-801G>A polymorphism.

In 6 out of 123 ERSD patients we found PCR (Polymerase Chain Reaction) products with two patterns of DNA fragments: 502bp/397bp and 449bp/397bp (Figure 1A). Sequencing of these fragments revealed two duplications in heterozygosity: an extra block composed by the 4th and 5th repetitions of the promoter region between the 5th and 6th repetitions, and an extra 52bp between the 4th and 5th repetitions, respectively (the latter was described in previous studies) [4]. In the remaining 117 patients, we identified two SNPs already described: c.-741G>A (Figure 1B.I–B.III) and c.-903T>G (Figure 1C.I,C.II). Moreover, we found a new SNP, the c.-801G>A (Figure 1D.I,D.II). The prevalence of each polymorphism and its allelic frequencies are presented on Table 1.

Heterozygosity for the c.-903T>G polymorphism did not influence the circulating levels of elastase (TT: 30.7 (21.2–41.1) ng/mL; TG: 28.7 (19.3–38.9) ng/mL; *p* = 0.673), neither none of the other evaluated variables (Table 1, supplementary data). Similarly, we did not find significant differences in plasma levels of elastase between the three genotypes associated with the polymorphism c.-741G>A (GG: 32.3 (23.7–40.2) ng/mL; GA: 27.9 (18.3–44.1) ng/mL; AA: 18.9 (17.2–20.4) ng/mL; *p* = 0.441) (Table 2, supplementary data). We also found a trend towards a decrease in the elastase/neutrophil ratio in ESRD patients homozygous for this polymorphism [GG: 8.7 ± 3.5; GA: 9.5 ± 6.2; AA: 3.4 ± 1.1; *p* = 0.074]. Moreover, we found that patients with the c.-741G>A allele, but not the GG genotype, had a dominant effect on leucocyte counts (GG: 6.2 ± 1.8 × 10^9^/L; GA: 6.2 ± 1.6 × 10^9^/L; AA: 8.8 ± 3.5 × 10^9^/L; *p* = 0.048), neutrophil counts (GG: 3.8 ± 1.3 × 10^9^/L; GA: 3.6 ± 0.9 × 10^9^/L; AA: 6.9 ± 3.7 × 10^9^/L; *p* < 0.001) and neutrophil/lymphocyte ratio (GG: 2.6 ± 1.2; GA: 2.3 ± 0.8; AA: 5.9 ± 4.5; *p* < 0.001) as well as levels on oxLDL (GG: 34.6 ± 9.5 U/L; GA: 33.9 ± 11.8 U/L; AA: 53.1 ± 25.5 U/L; *p* = 0.013) and serum albumin (GG: 3.9 ± 0.4 g/dL; GA: 4.0 ± 0.3 g/dL; AA: 3.5 ± 1.0 g/dL; *p* = 0.028) (Table 2, supplementary data). The different analyzed parameters associated with the new polymorphism c.-801G>A and the new extra block are presented in Table 3 - supplementary data. As elastase levels are influenced by different factors, it is difficult to relate their levels with the presence of heterozygosity. The patient with the polymorphism c.-801G>A presented values of elastase and elastase/neutrophil ratio similar to those obtained in total patients. Regarding the extra blocks, both patients have very different values in these parameters.

## 4. Conclusions

ESRD patients under dialysis present high elastase plasma levels, which has been associated with the rise in neutrophils, common in inflammatory processes and also associated with the hemodialysis procedure. The c.-741G>A polymorphism in the *ELANE* promoter seems to be associated with higher neutrophil counts, and therefore, with an enhanced inflammatory process that is usually associated with a poor outcome. Further studies with a larger population are required to confirm the influence of the GG genotype for c.-741G>A polymorphism in the inflammatory process and to assess the impact of the new mutation and the extra blocks described herein for the first time.

## Figures and Tables

**Table 1:** Prevalence and allele frequencies for each polymorphism identified in the studied end-stage renal disease (ESRD) patients.

	Polymorphism	Genotype	Number of Cases		Allelic Frequencies	
			N	%	Allele	%
**Previously Described**	c.-903T>G	TT	111	90.2	T	95.1
		TG	12	9.8		
					G	4.9
		GG	0	0.0		
	c.-741G>A	GG	84	68.3	G	82.9
		GA	36	29.3		
					A	17.1
		AA	3	2.4		
	Extra 52 pb	Wild type	119	96.7	Wild type	98.4
		Heterozygous	4	3.3		
					Extra 52 bp	1.6
		Homozygous	0	0.0		
**New**	c.-801G>A	GG	122	99.2	G	99.6
		GA	1	0.8		
					A	0.4
		AA	0	0.0		
	Extra block	Wild type	121	98.4	Wild type	99.2
		Heterozygous	2	1.6		
					Extra block	0.8
		Homozygous	0	0.0		

## Supplementary Materials

Supplementary materials can be accessed at: http://www.mdpi.com/2073-4425/7/5/17/s1.
