# Supplementary material for: Polymorphisms of the ELANE Gene Promoter Region in End-Stage Chronic Kidney Disease Patients
Source: Genes (Basel). 2016 Apr 29;7(5):17. doi: 10.3390/genes7050017 (PMC4880837; doi:10.3390/genes7050017)
Supplement: Supplementary file 1 [file genes-07-00017-s001.pdf]

Supplementary Materials: Polymorphisms of the *ELANE* Gene Promoter Region in End-Stage Chronic Kidney Disease Patients

Rafael Fernandes, Bruno Freitas, Vasco Miranda, Elísio Costa, Alice Santos-Silva and Elsa Bronze-da-Rocha

Table 1. Clinical and sociodemographic data, dialysis efficiency, hematological values and iron metabolism, inflammatory and nutritional markers for the polymorphism c.-903T>G.

Evaluated Variables	**Total Population (*n* = 123)**	**c.-903T>G TT (*n* = 111)**	**c.-903T>G TG (*n* = 12)**	***p* value**
**Clinical data and dialysis efficiency markers**				
**Age (years)**	65.3 ± 13.9	65.2 ± 13.5	66.4 ± 17.2	0.776
**Gender (men %)**	56.1	54.1	75.0	0.165
**CVC (n %)**	18 (14.6)	18 (16.2)	0 (0.0)	0.211
**AVF (n %)**	105 (85.4)	93 (83.8)	12 (100)	
**Diabetic patients (n %)**	45 (36.6)	39 (35.1)	6 (50.0)	0.353
**Time on dialysis (years)**	2.5 (1.2–5.2)	2.7 (1.2–5.3)	2.2 (1.1–5.1)	0.780
**URR (%)**	75.9 ± 6.3	75.9 ± 6.0	76.8 ± 8.4	0.627
**Kt/V**	1.5 ± 0.3	1.5 ± 0.3	1.5 ± 0.2	0.824
**Creatinine (mg/dL)**	8.3 ± 2.9	8.3 ± 2.9	8.7 ± 2.9	0.619
**Darbopoeitin (μg/kg/week)**	0.4 (0.2–0.7)	0.4 (0.2–0.8)	0.6 (0.2–0.7)	0.680
**Hematological data**				
**Hemoglobin (g/dL)**	11.8 ± 1.2	11.8 ± 1.4	11.9 ± 1.4	0.824
**Hematocrit (%)**	36.8 ± 4.5	36.8 ± 4.5	36.5 ±4.6	0.808
**Erythrocytes (×10^{12}/L)**	3.8 ± 0.5	3.8 ± 0.5	3.8 ± 0.5	0.975
**MCV (fL)**	95.8 ± 4.9	95.8 ± 5.0	95.0 ± 4.3	0.557
**MCH (pg)**	30.8 ± 2.1	30.7 ± 2.1	31.4 ± 1.9	0.352
**MCHC (g/dL)**	32.2 ± 1.2	32.1 ± 1.2	32.7 ± 1.1	0.148
**Reticulocytes (×10^{9}/L)**	50.5 ± 28.6	50.2 ± 27.1	53.9 ± 45.3	0.714
**RPI**	1.0 ± 0.6	1.0 ± 0.5	1.0 ± 0.9	0.754
**Leucocytes (×10^{9}/L)**	6.2 ± 1.8	6.2 ± 1.8	6.2 ± 1.8	0.965
**Neutrophils (×10^{9}/L)**	3.9 ± 1.4	3.8 ± 1.4	3.9 ± 1.2	0.921
**Lymphocytes (×10^{9}/L)**	1.6 ± 0.6	1.6 ± 0.6	1.6 ± 0.6	0.618
**Neutrophil/lymphocyte ratio**	2.6 ± 1.3	2.6 ± 1.3	2.8 ± 1.3	0.640
**Iron metabolism markers**				
**Ferro (mg/dL)**	43.3 ± 25.6	42.2 ± 24.2	53.8 ± 35.4	0.134
**Transferrina (mg/dL)**	177.6 ± 33.9	177.2 ± 34.2	181.7 ± 32.4	0.665
**Transferrin saturation (%)**	17.9 ± 11.4	17.3 ± 10.4	22.6 ± 18.7	0.134
**Ferritin (ng/mL)**	400.2 ± 157.5	399.7 ± 151.7	404.9 ± 212.3	0.914
**sTfR (nmol/L)**	23.9 ± 11.5	23.9 ± 11.3	24.8 ± 13.3	0.780
**Inflammatory related variables**				
**CRP (mg/dL)**	4.8 (2.2-13.3)	4.9 (2.2–14.4)	3.7 (1.9–8.9)	0.509
**IL-6 (pg/mL)**	2.2 (1.3–4.0)	2.1 (1.3–3.9)	2.3 (1.6-5.0)	0.551
**OxLDL (U/L)**	34.9 ± 11.0	34.9 ± 10.8	34.1 ± 13.1	0.805
**Elastase (ng/mL)**	30.5 (21.2–40.8)	30.7 (21.2–41.1)	28.7 (19.3–38.9)	0.673
**Elastase/neutrophil ratio**	8.8 ±4.5	8.9 ±4.6	8.0 ±3.2	0.513
**Nutritional markers**				
**Albumin (g/dL)**	3.9 ± 0.4	3.9 ± 0.4	4.0 ± 0.3	0.313
**BMI (Kg/m^{2})**	25.9 ± 4.6	25.9 ± 4.7	26.0 ± 3.5	0.947

AVF: arteriovenous fistula; BMI: body mass index; CRP: C-reactive protein; CVC: central venous catheter; *Kt/V:* dialysis clearance of urea; IL-6: interleukin 6; MCH: mean cell hemoglobin; MCHC: mean cell hemoglobin concentration; MCV: mean cell volume; oxLDL: oxidized low-density lipoprotein; RDW:

red cell distribution width; RPI: reticulocyte production index; s-TfR: soluble transferrin receptor; URR: urea clearance ratio by dialysis in the dialysis time/urea distribution volume.

Table 2. Clinical and sociodemographic data, dialysis efficiency, hematological values and iron metabolism, inflammatory and nutritional markers for the polymorphism c.-741G>A.

Evaluated Variables	**Total Population (*n* = 123)**	**c.-741G>A GG (*n* = 84)**	**GA (*n* = 36)**	**AA (*n* = 3)**	***p* value**
**Clinical data and dialysis efficiency markers**					
**Age (years)**	65.3 ± 13.9	66.6 ± 13.4	61.9 ± 15.0	68.4 ± 3.6	0.226
**Gender (men %)**	56.1	52.4	63.9	66.7	0.474
**CVC (n %)**	18 (14.6)	12 (14.3)	6 (16.7)	0 (0.0)	0.726
**AVF (n %)**	105 (85.4)	72 (85.7)	30 (83.3)	3 (100)	
**Diabetic patients (n %)**	45 (36.6)	33 (39.3)	10 (27.8)	2 (66.7)	0.267
**Time on dialysis (years)**	2.5 (1.2–5.2)	2.7 (1.2–5.2)	2.5 (1.3–7.4)	2.4 (2.0–X)	0.386
**URR (%)**	75.9 ± 6.3	76.3 ± 6.0	74.9 ± 6.9	77.8 ± 4.7	0.448
**Kt/V**	1.5 ± 0.3	1.5 ± 0.4	1.4 ± 0.2	1.7 ± 0.1	0.351
**Creatinine (mg/dL)**	8.3 ± 2.9	8.2 ± 2.9	8.7 ± 2.9	6.7 ± 3.1	0.396
**Darbopoeitin (μg/kg/week)**	0.4 (0.2–0.7)	0.4 (0.2–0.7)	0.5 (0.2–0.7)	0.4 (0.4–X)	0.848
**Hematological data**					
**Hemoglobin (g/dL)**	11.8 ± 1.2	11.7 ± 1.4	12.2 ± 1.2	11.5 ± 1.3	0.102
**Hematocrit (%)**	36.8 ± 4.5	36.4 ± 4.6	38.0 ± 4.1	34.3 ± 4.3	0.131
**Erythrocytes (×10^{12}/L)**	3.8 ± 0.5	3.8 ± 0.5	3.9 ± 0.4	3.5 ± 0.4	0.199
**MCV (fL)**	95.8 ± 4.9	95.4 ± 5.0	96.5 ± 4.7	98.5 ± 4.3	0.329
**MCH (pg)**	30.8 ± 2.1	30.5 ± 2.1	31.3 ± 2.0	32.6 ± 1.1	0.118
**MCHC (g/dL)**	32.2 ± 1.2	32.1 ± 1.1	32.3 ± 1.3	33.4 ± 0.5	0.162
**Reticulocytes (×10^9/L)**	50.5 ± 28.6	52.4 ± 30.2	45.4 ± 25.5	57.8 ± 4.1	0.445
**RPI**	1.0 ± 0.6	1.0 ± 0.6	0.9 ± 0.6	1.1 ± 0.2	0.601
**Leucocytes (×10^9/L)**	6.2 ± 1.8	6.2 ± 1.8	6.2 ± 1.6	8.8 ± 3.5	0.048
**Neutrophils (×10^9/L)**	3.9 ± 1.4	3.8 ± 1.3	3.6 ± 0.9	6.9 ± 3.7	<0.001
**Lymphocytes (×10^9/L)**	1.6 ± 0.6	1.6 ± 0.5	1.8 ± 0.7	1.3 ± 0.3	0.250
**Neutrophil/lymphocyte ratio**	2.6 ± 1.3	2.6 ± 1.2	2.3 ± 0.8	5.9 ± 4.5	<0.001
**Iron metabolism markers**					
**Ferro (mg/dL)**	43.3 ± 25.6	42.6 ± 26.3	44.3 ± 25.2	50.0 ± 6.2	0.858
**Transferrina (mg/dL)**	177.6 ± 33.9	177.1 ± 34.5	181.1 ± 30.0	150.0 ± 61.2	0.308
**Transferrin saturation (%)**	17.9 ± 11.4	17.5 ± 11.1	17.9 ± 11.7	28.3 ± 17.4	0.278
**Ferritin (ng/mL)**	400.2 ± 157.5	406.1 ± 167.4	382.4 ± 135.6	448.1 ± 131.4	0.655
**sTfR (nmol/L)**	23.9 ± 11.5	24.4 ± 12.1	23.1 ± 10.2	20.8 ± 7.4	0.754
**Inflammatory related variables**					
**CRP (mg/dL)**	4.8 (2.2–13.3)	3.9 (2.1–12.8)	5.8 (2.7–15.5)	3.5 (1.9–X)	0.490
**IL-6 (pg/mL)**	2.2 (1.3–4.0)	2.1 (1.3–3.7)	2.1 (1.5–4.3)	3.5 (0.9–X)	0.519
**OxLDL (U/L)**	34.9 ± 11.0	34.6 ± 9.5	33.9 ± 11.8	53.1 ± 25.5	0.013
**Elastase (ng/mL)**	30.5 (21.2–40.8)	32.3 (23.7–40.2)	27.9 (18.3–44.1)	18.9 (17.2–20.4)	0.441
**Elastase/neutrophil ratio**	8.8 ±4.5	8.7 ±3.5	9.5 ± 6.2	3.4 ± 1.1	0.074
**Nutritional markers**					
**Albumina, g/dL**	3.9 ± 0.4	3.9 ± 0.4	4.0 ± 0.3	3.5 ± 1.0	0.028
**BMI, Kg/m^2)**	25.9 ± 4.6	25.9 ± 4.3	26.0 ± 5.2	26.3 ± 6.9	0.981

AVF: arteriovenous fistula; BMI: body mass index; CRP: C-reactive protein; CVC: central venous catheter; *Kt/V:* dialysis clearance of urea; IL-6: interleukin 6; MCH: mean cell hemoglobin; MCHC: mean cell hemoglobin concentration; MCV: mean cell volume; oxLDL: oxidized low-density lipoprotein; RDW: red cell distribution width; RPI: reticulocyte production index; s-TfR: soluble transferrin receptor; URR: urea clearance ratio by dialysis in the dialysis time/urea distribution volume.

Table 3. Clinical and sociodemographic data, dialysis efficiency, hematological values and iron metabolism, inflammatory and nutritional markers for the polymorphism c.-801G>A (n = 1) and extra bock (n = 2).

Evaluated Variables	**c.-801G>A (n = 1)**	**Extra block (Case 1)**	**Extra block (Case 2)**
**Clinical data and dialysis efficiency markers**			
**Age (years)**	66.6	67.3	62.7
**Gender**	Female	Female	Male
**Vascular access**	AVF	AVF	CVC
**Diabetic patient**	Yes	Yes	No
**Time on dialysis (years)**	0.8	2.9	0.2
**URR (%)**	75	71.0	64.6
**Kt/V**	1.4	1.20	1.0
**Creatinine (mg/dL)**	6.9	9.4	3.2
**Darbopoeitin (μg/kg/week)**	0.5	0.5	0.7
**Hematological data**			
**Hemoglobin (g/dL)**	12.2	11.9	11.5
**Hematocrit (%)**	36.4	35.8	35.5
**Erythrocytes (×10^{12}/L)**	4.0	3.6	4.1
**MCV (fL)**	90.1	98.4	87.2
**MCH (pg)**	ND	32,7	28.3
**MCHC (g/dL)**	33.5	33.2	32.4
**Reticulocytes (×10^9/L)**	105.0	72.8	40.7
**RPI**	2.1	1.6	0.8
**Leucocytes (×10^9/L)**	4.0	5.0	6.6
**Neutrophils (×10^9/L)**	2.9	3.7	4.4
**Lymphocytes (×10^9/L)**	0.9	1.0	1.6
**Neutrophil/lymphocyte ratio**	3.4	3.9	2.7
**Iron metabolism markers**			
**Ferro (mg/dL)**	27	38	35
**Transferrina (mg/dL)**	143	180	191
**Transferrin saturation (%)**	13.4	14.9	13.0
**Ferritin (ng/mL)**	361.4	443.7	484.5
**sTfR (nmol/L)**	38.4	14.0	34.2
**Inflammatory related variables**			
**CRP (mg/dL)**	0.31	2.2	31.3
**IL-6 (pg/mL)**	1.8	1.8	4.4
**OxLDL (U/L)**	26.5	38.4	33.4
**Elastase (ng/mL)**	32.2	20.3	47.8
**Elastase/neutrophil ratio**	11.1	5.5	10.9
**Nutritional markers**			
**Albumina, g/dL**	3.4	4.1	3.8
**BMI, Kg/m^2**	24.1	30.1	32.8

AVF: arteriovenous fistula; BMI: body mass index; CRP: C-reactive protein; CVC: central venous catheter; *Kt/V*: dialysis clearance of urea; IL-6: interleukin 6; MCH: mean cell hemoglobin; MCHC: mean cell hemoglobin concentration; MCV: mean cell volume; oxLDL: oxidized low-density lipoprotein; RDW: red cell distribution width; RPI: reticulocyte production index; s-TfR: soluble transferrin receptor; URR: urea clearance ratio by dialysis in the dialysis time/urea distribution volume.
